# Structural domains of SARS-CoV-2 nucleocapsid protein coordinate to compact long nucleic acid substrates

**DOI:** 10.1093/nar/gkac1179

**Published:** 2022-12-19

**Authors:** Michael Morse, Jana Sefcikova, Ioulia Rouzina, Penny J Beuning, Mark C Williams

**Affiliations:** Department of Physics, Northeastern University, Boston, MA, USA; Department of Chemistry and Chemical Biology, Northeastern University, Boston, MA, USA; Department of Chemistry and Biochemistry, Ohio State University, Columbus, OH, USA; Department of Chemistry and Chemical Biology, Northeastern University, Boston, MA, USA; Department of Physics, Northeastern University, Boston, MA, USA

## Abstract

The SARS-CoV-2 nucleocapsid (N) protein performs several functions including binding, compacting, and packaging the ∼30 kb viral genome into the viral particle. N protein consists of two ordered domains, with the N terminal domain (NTD) primarily associated with RNA binding and the C terminal domain (CTD) primarily associated with dimerization/oligomerization, and three intrinsically disordered regions, an N-arm, a C-tail, and a linker that connects the NTD and CTD. We utilize an optical tweezers system to isolate a long single-stranded nucleic acid substrate to measure directly the binding and packaging function of N protein at a single molecule level in real time. We find that N protein binds the nucleic acid substrate with high affinity before oligomerizing and forming a highly compact structure. By comparing the activities of truncated protein variants missing the NTD, CTD, and/or linker, we attribute specific steps in this process to the structural domains of N protein, with the NTD driving initial binding to the substrate and ensuring high localized protein density that triggers interprotein interactions mediated by the CTD, which forms a compact and stable protein-nucleic acid complex suitable for packaging into the virion.

## INTRODUCTION

**Figure 1.**
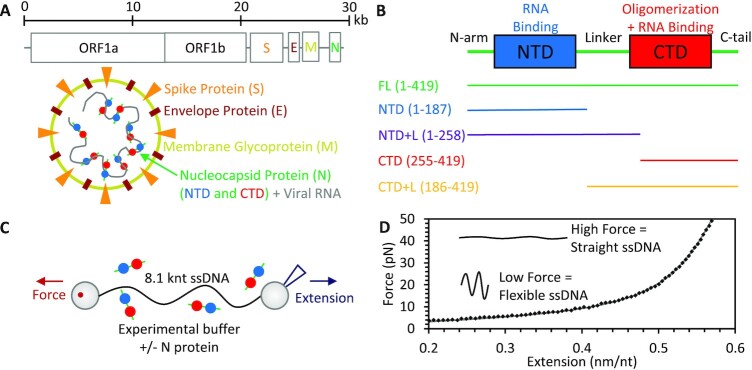
Overview of SARS-CoV-2 N protein and experimental system. (**A**) The SARS-CoV-2 virion is constructed from four structural proteins. The nucleocapsid protein (N) binds and packages the viral RNA genome inside the viral particle. (**B**) The N protein consists of two ordered domains, NTD (blue) and CTD (red), separated by an IDR linker and flanked by additional IDRs at the N and C termini (green). In addition to the full-length (419 aa) N protein, four variants were also expressed and purified containing either the NTD or CTD alone or with the linker attached. (**C**) In optical tweezers experiments, a single long (8.1 knt) ssDNA substrate is isolated by tethering between two functionalized beads. One bead is held by a stationary dual beam counter-propagating optical trap while the other is held by a micropipette tip. The tip is moved with nm resolution to control the extension of the substrate while the deflection of the laser trap is measured to determine the tension across the ssDNA. (**D**) Stretching protein-free ssDNA (black points) is well fit by the freely jointed chain (FJC) model (gray line).

Severe acute respiratory syndrome coronavirus 2 (SARS-CoV-2), the virus responsible for the COVID-19 pandemic, encodes four structural proteins, which comprise the infectious viral particle (1). While the spike (S), membrane (M), and envelope (E) proteins comprise the outer shell of the virion at the interface with the external environment, the nucleocapsid (N) protein in complex with the ∼30 kb viral RNA (vRNA) genome is contained in the virion's interior (Figure 1A). Thus, the primary role of N protein is to package the vRNA inside the assembled viral particle (2,3). This necessarily requires a large degree of compaction for vRNA (∼16 μm linear length) to fit inside a virion less than 100 nm in diameter. Besides packaging, N protein has been shown to function later in the viral life cycle, including in regulation of transcription and vRNA synthesis in infected cells (4,5). N protein is highly conserved among coronaviruses, with the 46 kDa SARS-CoV-2 N protein's 419 amino acid sequence 90% homologous to that of SARS-CoV-1 (6). The essential and conserved nature of N protein has made it a popular target for both PCR and antigen testing and a potential target for therapies (7–9) and vaccination (10).

N protein binds single stranded nucleic acid (NA) substrates with high affinity, including non-specific RNA and DNA (6,11). The structure of N protein consists of two globular, ordered domains, with resolved structures similar to other coronaviruses (11–14), and three intrinsically disordered regions (IDRs) (Figure 1B) (15). The ordered N terminal domain (NTD) binds RNA (12,14,16,17) and is sometimes referred to as the RNA binding domain (RBD), though N protein is highly basic with patches of positively charged regions throughout the protein capable of NA binding (18). The ordered C terminal domain (CTD) self-associates to form stable dimers in solution (6,9,13,19–22), and higher order oligomeric states have also been predicted and observed (20,23–25). The IDR between the domains acts as a linker, limiting interaction between the NTD and CTD and enabling potential independent function of the two domains (26). This linker has an arginine/serine rich region that can undergo post translational phosphorylation, which can in turn alter protein function (27,28). IDRs are also present at each of the protein termini, referred to as the N-arm and C-tail, which can also contribute to RNA binding (18). Additionally, coronavirus N proteins have the ability to unwind RNA and DNA duplexes (16,29,30). While protein-free vRNA folds into a complex structure consisting of stems, bulges, loops, and pseudoknots, which were theoretically predicted and experimentally measured (31,32), it is unclear to what degree secondary structure is preserved when the vRNA is protein saturated, such as the case for N protein-mediated packaging.

One potential method to distinguish the particular function of each of these domains is to compare the functions of truncated protein variants in an *in vitro* assay. That is, proteins that lack either the NTD or CTD (Figure 1B) are expressed and purified (see Materials and Methods and Supplementary Figure S1 for full sequence) and their NA binding and packaging functions are measured and compared. In this study, we isolate a single long (8.1 knt) ssDNA substrate using an optical tweezers system (Figure 1C), which allows us to measure the protein–ssDNA interactions in real time at a single molecule level. Note, optical tweezers typically require DNA substrates rather than pure RNA as the initial tethering procedure requires a stiffer double stranded substrate and labeled base pairs at the ends (biotinylated here). However, the biophysical polymer properties (length and stiffness) of ssDNA and ssRNA are very similar (33,34), such that the work required to compact the two substrates into the same conformation would be equivalent. Furthermore, studies incubating SARS-CoV-2 N protein and other related coronavirus nucleocapsid proteins in bulk solutions of various RNA and DNA substrates have measured similar non-specific binding affinities for both ssDNA and ssRNA (16,29,30,35).

Optical tweezers are an ideal system to study NA packaging, as a single nucleic acid molecule's structural conformation is directly measured in physiologically relevant buffer conditions in the presence of unlabeled proteins. Additionally, by measuring substrate extension as a function of tension, the structural dynamics of NA-protein complexes can be resolved, and this conformation can be disrupted or stabilized by adjusting applied force. This reveals novel insights into the dynamics of the N protein-vRNA complex, which has not been resolved for full length proteins or oligomeric protein. We observe ssDNA compaction in the presence of N protein, with the kinetics and amplitude of this compaction and the stability of these structures dependent on which structural domains are present in the protein. Thus, we do not just observe the final equilibrated state of the NA-protein complex, but also intermediate states associated with cooperative and non-cooperative binding modes. Our results reveal a kinetic pathway that suggests a mechanism by which free N protein saturates viral RNA in the cytoplasm of infected cells and subsequently packages the viral genome in a manner that allows encapsulation into the viral particle.

## MATERIALS AND METHODS

### Plasmid Synthesis and Protein Expression and Purification

Unless noted, reagents were from Fisher Scientific (Hampton, NH). The pET11T plasmid carrying the codon-optimized gene sequence of full-length (FL) N protein, 419 amino acids of NCAP_SARS2 (Uniprot ID P0DTC9) (full sequence, Supplementary Figure S1), and encoding ampicillin (AMP) resistance was synthesized by GenScript Biotech (Piscataway, NJ). Mutagenic and sequencing primers were purchased from Eurofins Genomics (Louisville, KY). Sequences of full-length N protein and its truncated variants were validated by sequencing at Eton Bioscience (Charlestown, MA). The gene encoding the N protein and variants was designed to have an NheI recognition site at the 5′ end following sequences coding for the His-tag and the recognition site for PreScission protease. Truncated variants containing the globular N-terminal domain only without (NTD, amino acids 1–191) and with the flexible linker (NTD + L, amino acids 1–262) were generated via site-directed mutagenesis using mutagenic primers with stop codons at the amino acid positions 187 and 258, respectively. The truncated variants containing the globular C-terminal domain only without (CTD, amino acid 254–419) and with the flexible linker (CTD + L, amino acid 186–419) were produced by site-directed mutagenesis using mutagenic primers to introduce NheI recognition sites at the amino acid positions 185–186 and 254–255, respectively. The resulting plasmids were digested by NheI restriction enzyme (New England Biolabs (NEB), Ipswich MA) for 4 h at 37°C. Cleaved products were verified on 1% agarose gel and ligated by T4 DNA ligase (NEB) overnight at room temperature (RT). Ligated plasmid products were verified on 1% agarose gel. DH5α competent cells were transformed with plasmids encoding the constructs, which were validated by sequencing (Eton Biosciences).

BL21 competent cells carrying plasmid pLysS encoding chloramphenicol (CM) resistance were transformed with plasmids expressing N protein and variants. A single colony was used to inoculate 50 ml of Luria broth media supplemented with 100 μg/ml AMP, 25 μg/ml CM and 1% d-glucose, and incubated with shaking at 37°C overnight. This culture was then used to inoculate 1 l of Luria broth media supplemented with 100 μg/ml AMP, 25 μg/ml CM, which was grown with shaking at 37°C until OD_600_ reached at least 0.8. Protein expression was then induced with 0.25 mM IPTG (RPI, Mount Prospect, IL) at RT overnight. Cells were harvested by centrifugation at 5000 × *g*, for 15 min, 4°C and stored at -80°C.

Purification of each protein began by adding His buffer A/lysis buffer (50 mM HEPES pH 7.5, 500 mM NaCl, 50 mM imidazole, 5 mM β-mercaptoethanol, 5% glycerol) to the frozen cell pellet up to 40 ml, 50 μl of freshly made 10 mg/ml PMSF in isopropanol, a half tablet of cOmplete mini protease inhibitor cocktail (Roche, Basel Switzerland), and lysozyme. Cells were placed on ice and thawed overnight at 4°C, at which point the cell mixture was divided into two tubes and His buffer A/lysis buffer was added to 40 ml. Both tubes were sonicated in 15 s on and off intervals for 5 min each followed by addition of DNase I, and then incubated on a rocker for 1 h at 4°C. The resuspended cell pellet was placed at −80°C for 30 min and then in a 37°C water bath for 25 min. Cell debris was removed by centrifugation at 12 000 × *g* for 1 h at 4°C. The supernatant was loaded on a HisTrap column (Cytiva Life Sciences, Marlborough, MA) using an Akta FPLC (Cytiva Life Sciences) and protein was eluted with His buffer B (50 mM HEPES pH 7.5, 500 mM NaCl, 500 mM imidazole; 5 mM β-mercaptoethanol, 5% glycerol). The His-tag on N protein was cleaved by PreScission protease in a 10:1 (N:PreScission) ratio during dialysis in 2 L dialysis buffer (50 mM HEPES pH 7.5, 300 mM NaCl, 5 mM β-mercaptoethanol, 5% glycerol) at 4°C overnight. The cleavage reaction was loaded on the HisTrap column and collected during the flow through period of the method with His buffer B300 (50 mM HEPES pH 7.5, 300 mM NaCl, 500 mM imidazole; 5 mM β-mercaptoethanol, 5% glycerol). Protein was concentrated using Vivaspin 6 10 kDa MWCO concentrators (Sartorius, Bohemia, NY) and buffer exchanged to the storage buffer (20 mM HEPES pH 7.5, 150 mM NaCl, 1 mM DTT, 5% glycerol). Each protein preparation was stored in single-use aliquots at -80°C.

### Manipulation of ssDNA by optical tweezers

The DNA substrate was prepared by double digesting the 8.1 kb transfer plasmid pBACgus11 (gift from Borja Ibarra, IMDEA Nanociencia) with restriction enzymes BamHI and SacI (NEB) then ligating biotinylated oligos (Integrated DNA Technologies) to the resulting overhangs (36). Gel purification was used to remove excess biotinylated oligos and the labeled DNA was diluted to a concentration of 10 ng/ml in our experimental buffer (buffer E, 150 mM NaCl, 10 mM HEPES, pH 7.5). DNA was flowed into the sample chamber and tethered between two streptavidin coated beads, one held in a stationary dual beam counter propagating optical trap and the other held by a glass micropipette tip. The tip is moved by a piezo electric stage, controlling the distance between the beads and thus the DNA extension with 1 nm precision, while bright field images of the two beads are simultaneously captured to independently measure inter-bead distance and correct for long term thermal drift in the system (37,38). The deflection of the laser trap was also measured to determine the force applied to the trapped bead (0.1 pN resolution at ∼40 Hz) and thus the tension along the DNA. By extending the DNA in the presence of 10 mM NaOH, base pairing was disrupted allowing the unlabeled strand to dissociate, leaving an entirely ssDNA tethered between the two beads (Figure 1C). The sample chamber was flushed with buffer E and the quality of the ssDNA substrate was assured by comparing its force extension curve (FEC) for forces ≥5 pN (where most secondary structure is destabilized for this substrate) with the freely jointed chain (FJC) polymer model before each experiment (Figure 1D).

### Protein binding and ssDNA compaction experiments

Protein binding to and compacting the ssDNA substrate was measured in real time using a force feedback loop to maintain constant tension along the substrate. When the measured tension on the ssDNA was less than the target force (typically 10 pN unless otherwise noted), the ssDNA extension was incrementally increased until the target force was achieved. The extension was similarly decreased when the measured force was above the target force, resulting in <0.1 pN deviations in ssDNA tension during constant force measurement. N protein diluted to a set concentration in buffer E was flowed into the sample, allowing binding to the isolated ssDNA. The substrate's change in extension was measured in real time at ∼40 Hz. After the ssDNA–protein complex reached equilibrium, protein was removed from the sample by flowing in protein free buffer, with the resulting change in substrate extension again measured in real time. Alternatively, the extension of the ssDNA–protein complex was measured by stretching the substrate from starting extension of ∼0.2 nm/nt (less than half the length of fully extended ssDNA, 0.55 nm/nt) until a force of 80 pN was achieved. This stretching was performed either directly after incubation with N protein for 100 s, or after incubation with N protein for 100 s followed by dissociation of bound protein into protein free buffer for 200 s. All experiments were performed as *N* ≥ 3 experimental replicates on different substrates to ensure reproducibility, and plotted error bars are standard error of the mean.

## RESULTS

### Full length N protein compacts nucleic acid substrate in multiple steps

**Figure 2.**
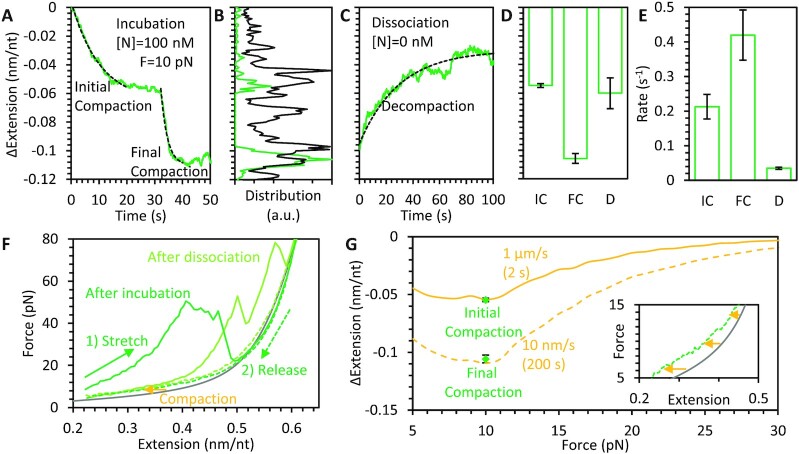
Compaction of ssDNA by FL N protein. (**A**) ssDNA held at 10 pN constant tension is incubated and immediately bound and compacted by 100 nM N protein. ssDNA extension does not decrease monotonically, but temporarily pauses before continuing to compact after a variable time delay. Both compaction steps are well fit by exponential equations (dashed lines). (**B**) Instantaneous values of ssDNA compaction during protein incubation for both single experiment (panel A data, green) and all experiments summed (*N* = 10, black) show peaks at partial compaction, indicating consistent pausing before reaching full compaction. (**C**) After 100 s N protein incubation, free protein is removed from the sample, allowing for protein dissociation without replacement, resulting in partial decompaction (green, same experiment as A) well fit by an exponential dependence (dashed line). Amplitudes (**D**) and rates (**E**) of exponential fits to initial (IC) and final compaction (FC) during incubation (panel A) and decompaction (D) during dissociation (panel C) are averaged for all experiments (*N* = 10). Compaction amplitudes of 0.05 and 0.1 nm/nt are consistently observed after the first compaction step and last compaction step, respectively, in agreement with the two largest peaks in the instantaneous compaction histogram (panel B). The final compaction step is twice as fast as the initial step on average. Decompaction consistently occurs over a 50 s timescale but only results in partial decompaction. (**F**) The ssDNA–protein complex is stretched immediately after protein incubation (solid green), exhibiting a large degree of substrate compaction (reduced extension). At high force, these protein-mediated compacted structures are destabilized, resulting in ssDNA extending to its protein-free length (gray line, FJC fit from Figure 1D). Decreasing the substrate extension to its original value (dashed green) displays reduced, but non-zero, compaction. Stretching the ssDNA after 200 s dissociation (light green) instead of directly after incubation (dark green) exhibits reduced but non-zero compaction, consistent with partial dissociation. (**G**) The decrease in extension for ssDNA–protein complexes after stretching (inset shows magnified view of panel F) is calculated as a function of force and averaged over *N* = 5 experiments. The decrease in extension is minimal at high force but increases as force is reduced. When the substrate extension is quickly reduced (1 μm/s, 2 s timescale), the decrease in extension at 10 pN is equal to the initial compaction step during incubation (panel A). When the ssDNA substrate extension is slowly reduced (10 nm/s, 200 s timescale), the decrease in extension is larger and comparable to final compaction.

Experiments to assess binding and compaction were first performed using FL N protein. A single ssDNA molecule was isolated and manipulated by its ends, such that we controlled its end-to-end extension and the resulting tension across its backbone. While ssDNA can form secondary structure that substantially reduces its end-to-end extension, increasing applied force destabilizes these structures. The force required to remove secondary structure depends on the exact sequence (hairpin length, GC content, mismatches, etc.), but we experimentally confirmed that this substrate is mostly linear at forces ≥ 5 pN, based on agreement with predicted polymer behavior (Figure 1D). Since protein binding is typically dependent on substrate tension, we fixed the ssDNA tension (while allowing extension to vary) while incubating with N protein diluted to a concentration of 100 nM in buffer E (Figure 2A). Unless otherwise noted, all experiments were performed at physiologically relevant salt concentrations (150 mM NaCl). As discussed in more detail later, we set the tension to 10 pN as lower values resulted in complete collapse of the substrate (end-to-end extension became negligible and the two tethering beads came into contact). Immediately after the ssDNA was exposed to N protein, the substrate length began to contract on a ∼5 s timescale. The length change followed a simple exponential pattern that formed an asymptote at ∼0.05 nm/nt reduction in length (approximately 1/8th the extended length of bare ssDNA held at 10 pN), which is consistent with free proteins binding to available binding sites at a constant rate. While the exact binding site size (nt occupied per protein) of N protein is not definitively known, this general model does not require a defined site size, but instead that any length of substrate sufficiently large to accommodate an additional protein without steric prohibition acts as a potential binding site. After a variable time delay (order of 10 s), however, we observed a secondary, fast compaction event, which approximately doubled the total degree of substrate compaction (∼0.1 nm/nt reduction in length). This multistep compaction process is observed for all replicate experiments performed (*N* = 10) and can be alternatively visualized by a histogram of all instantaneous measurements of ssDNA compaction during incubation (Figure 2B), both for individual experiments and all experiments combined, which shows two large peaks centered around 0.05 and 0.1 nm/nt. In contrast, if N protein bound and compacted the ssDNA in a single step, the substrate extension would quickly move through the minor compaction regime and the histogram would only show a peak at the final equilibrium length.

After the ssDNA–protein complex and its extended length were equilibrated (100 s), free protein was removed from the sample (Figure 2C). During this phase of the experiment, no additional protein can bind the ssDNA and all changes in ssDNA–protein conformation must be due to the dissociation of previously bound protein. We observe that the substrate partially decompacts during this time, but never reaches the original length of protein-free ssDNA on the timescale of our experiments (100s of seconds). Exponential functions are fit during dissociation, and the amplitudes and rates associated with these fits are averaged over all experiments (Figure 2D, E). Note, some experiments displayed a small additional compaction step between the initial and final steps (Supplementary Figure S2), which is responsible for the small middle peak in the compaction histogram, and these events were not averaged with either the initial or final compaction data. The ssDNA compaction after the initial and final steps are closely clustered around 0.05 and 0.1 nm/nt respectively, in agreement with the peaks in the compaction histogram. Additionally, the final compaction step is twice as fast on average (0.4 s^−1^) as the initial step (0.2 s^−1^), indicating a different mechanism is likely responsible for the two different modes of compaction. In comparison, decompaction due to dissociation occurs over a timescale of ∼50 s. This order of magnitude difference in rate suggests that the 100 nM N protein concentration used in these experiments should be sufficient to saturate the ssDNA. That is, since free protein binds to available binding sites ∼10X faster than bound protein dissociates from binding sites, ∼90% of available ssDNA binding sites should be occupied at equilibrium. Thus, the secondary compaction observed during incubation cannot be explained by simply additional protein binding and compacting the substrate. Rather, after the first decrease in extension, the ssDNA stops compacting as it becomes saturated with protein, and the second extension decrease must be due to a stochastically triggered highly cooperative reorganization of the ssDNA-protein complex.

We also probed the structure of the ssDNA-protein complex by rapidly extending it at a constant rate (1 μm/s) while simultaneously measuring substrate tension. The resulting force-extension curve (FEC) is compared to that of ssDNA alone (Figure 2F). Even when the ssDNA saturated with N protein was held at a fraction of its extended length, significant tension was maintained (>5 pN). This tension could not be relieved by further reduction in extension without the two tethering beads eventually colliding, consistent with the inability to perform constant force experiments at fixed tensions <10 pN. This is also consistent with the N protein packaging function, as the final compacted RNA substrate must be <100 nm in diameter to fit inside the viral particle. When the extension of the ssDNA substrate was increased, its tension immediately increased as well, compared to bare ssDNA, which greatly extends at low force as it is straightened. This indicates that the bound protein removed all slack from the ssDNA polymer chain. The FEC also shows greatly decreased extension compared to bare ssDNA at a given force, confirming the compaction observed during the preceding constant force experiment. While applied force typically increased with increased extension (positive slope in FEC), we also observed large decompaction events in which the tension along the ssDNA–protein complex suddenly decreased (negative slope in FEC). This decompaction occurred predominantly at high applied forces, and at the highest force applied, the length of the substrate approached that of bare ssDNA, indicating nearly all compacting structures had been removed.

The stretching curves of N protein-compacted ssDNA are highly variable. Sawtooth patterns of high force-induced rips releasing large variable lengths of substrate, which are irreproducible even for multiple stretches of the same substrate, indicate multiple long-range contacts within the relaxed N protein–ssDNA complex that can be removed by force. Some degree of experimental variability is also due to the stochastic nature of contact breaking as force is increased, with nucleic acid secondary structures or complexes randomly disrupted at higher or lower forces. A small degree of compaction remains after stretching, however, resulting in a slightly shorter (than protein-free ssDNA) complex when force is lowered. If the extension is lowered rapidly (1 μm/s), such that the tension is relieved in ∼2 s, minimizing changes in the complex structure, we observe a compaction of ∼0.05 nm at 10 pN (Figure 2G). This value is within error of both the average initial compaction during incubation and the average compaction remaining after partial protein dissociation. In contrast, slowly lowering complex extension (200 s timescale), allowing for reorganization, results in ∼0.1 nm/nt compaction at 10 pN, in agreement with the final compacted state observed during incubation. Note, unlike the stretching curves, the relaxation curves were smooth and reproducible, allowing the averaging of N = 5 experimental replicates to obtain average complex compactions as a function of force. These results indicate that while high force can eliminate higher levels of N protein-mediated ssDNA compaction, N protein remains bound in its initial binding state with only short-range protein/protein and protein/ssDNA interactions remaining.

Both this protein-mediated compaction at low ssDNA tension/extension and the spontaneous decompaction under high applied force can alternatively be visualized in a force jump experiment (Supplementary Figure S2E, F). When the tension on the substrate was suddenly increased to a high (≥20 pN force) constant force, decompaction of the ssDNA–protein complex was observed on a <10 s timescale in several stochastic, discrete steps. In contrast, when the substrate tension was suddenly decreased to 10 pN, a rapid compaction (similar in amplitude and rate to the second compaction event after protein saturation) occurred immediately and reproducibly between experiments.

**Figure 3.**
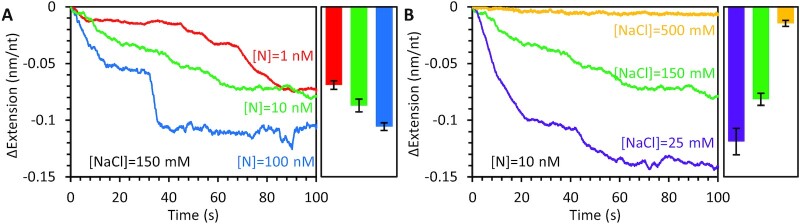
N protein binding and compaction at varying protein and salt concentrations. (**A**) Significant ssDNA compaction is observed for N protein concentrations as low as 1 nM. Low protein concentration requires a longer timescale to achieve full compaction and the two steps of compaction observed at high concentration are no longer individually resolvable. Right bar graph shows average extension change at the end of N protein incubation for each condition. (**B**) The degree of N protein-mediated ssDNA compaction is altered by the salt concentration of the experimental buffer, with very high NaCl concentrations inhibiting binding and compaction. Error bars represent standard error for *N* = 10 ([NaCl] = 150 mM, [N] = 100 nM) or *N* = 3 (all other conditions) experimental replicates.

We repeated the binding experiment with lower concentrations of free N protein (Figure 3A) and observed that compaction occurred over a longer timescale, consistent with binding initially occurring in a diffusion-limited manner. We still observed significant substrate compaction, however, at concentrations as low as 1 nM, indicating N protein binds ssDNA with high affinity. The kinetic profiles showed a different shape, lacking a defined pause at moderate compaction. This is expected for a multistep reaction, as being able to observe the steps separately requires the first step to occur at a much faster rate than subsequent steps, otherwise the steps occur concurrently instead of in series. For this system specifically, lower concentrations of N protein reduce the rate of initial binding, such that some regions of the ssDNA–protein complex can begin to compact locally before the entire substrate is saturated with protein. Another possibility is that the lower concentrations of N protein result in fewer dimeric and more monomeric proteins in solution. While the local concentration of protein along the binding substrate is much higher than in solution, thereby enabling the interprotein interactions required for oligomerization, compaction may require more time as the N proteins must undergo more degrees of oligomerization after binding when starting as monomers in solution. However, since N protein is highly expressed in host cells (>μM), our higher concentration N protein data, where N protein is likely dimeric in solution, is more representative of N protein function during viral replication. We also tested binding and compaction at much higher (500 mM NaCl) and lower (25 NaCl) salt concentrations (Figure 3B). As expected, low salt resulted in faster, more complete compaction, while high salt inhibited compaction. This result indicates that initial N protein binding to ssDNA and/or interprotein interactions required for the compacted form of the protein–ssDNA complex are at least partially electrostatic in nature.

### NTD protein variants bind noncooperatively, and compact nucleic acids similarly to the first step of compaction by full length N protein

**Figure 4.**
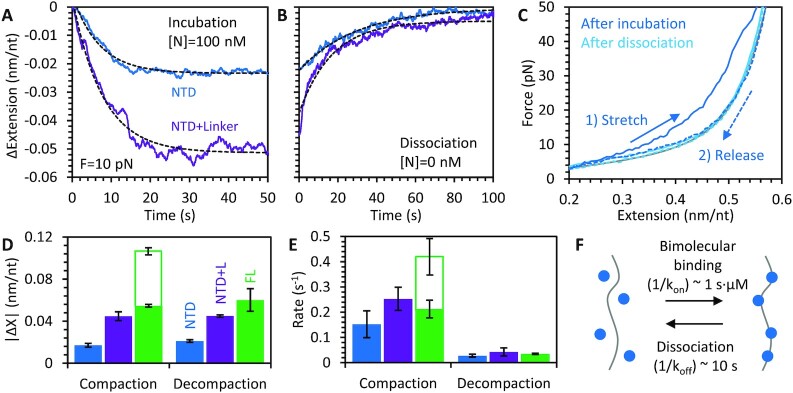
Compaction of ssDNA by NTD variant proteins. (**A**) Incubation of ssDNA held at 10 pN with N protein is repeated with NTD only (blue) and NTD plus linker (purple) protein. ssDNA compaction occurs on a single fast timescale well fit by an exponential dependence (dashed line). (**B**) Removing free protein from the sample results in full protein dissociation, with the ssDNA returning to its original conformation. (**C**) Stretching the ssDNA–protein complex after incubation (blue) shows slight compaction. After dissociation (light blue), the substrate follows the curve of protein free ssDNA (gray), confirming that all protein-compacted structures are removed during dissociation. The amplitude (**D**) and rate (**E**) of compaction during N protein incubation and decompaction in the absence of free protein are averaged over *N* = 3 experiments for both NTD protein variants and compared to those of FL protein (Figure 2). The rate of compaction for the NTD variants is comparable to the initial compaction of the FL protein (solid green bar), but slower than the final compaction step (hollow green bar). The NTD plus linker variant causes twice the ssDNA compaction as the NTD only variant, enabling compaction of the ssDNA similar to the final compaction observed for FL protein. (**F**) Model of NTD binding to nucleic acid substrate as a simple bimolecular reversible binding process.

Binding and compaction experiments were repeated using N protein truncations missing the CTD and disordered C-tail. Note, while we refer to these variants as NTD or NTD + L (with or without the IDR linker) for simplicity, these variants also contain the disordered N terminus, which assists in RNA binding (26), in addition to the ordered domain. During incubation of NTD with the ssDNA substrate held at constant 10 pN tension (Figure 4A), compaction occurs in a single step (i.e. no secondary compaction step is observed as in the case of FL protein). While the compaction caused by the NTD variant that includes the linker is similar to that of the initial compaction of the FL protein (∼0.05 nm/nt reduction in length), the NTD variant without the linker compacts the ssDNA much less (∼0.02 nm/nt reduction in length). For both variants, once free protein was removed, the ssDNA fully decompacted, returning to its original length, indicating all protein fully dissociated from the ssDNA (Figure 4B). Both the compaction during incubation and decompaction during dissociation are well fit by a single rate exponential decay, consistent with NTD variants binding the ssDNA substrate in a reversible, non-cooperative manner.

Stretching the ssDNA–protein complex formed using the NTD only N protein variant yielded similar results as the constant force measurements (Figure 4C). The FEC obtained directly after incubation showed reduced compaction (as compared to FL protein) that was disrupted at high force. Furthermore, stretching after 200 s of dissociation showed that the substrate was now completely protein-free ssDNA, closely following the FJC model. The amplitudes and rates of ssDNA compaction during incubation with NTD variants are compared to the initial compaction event observed for FL protein (Figure 4D, E). The NTD variant that lacks the linker compacts the ssDNA much less than the NTD + L and FL N proteins. In contrast, the rates of compaction and decompaction observed for all three protein variants are similar, and comparable to the initial compaction observed for FL protein. Our results with the NTD only N protein variants are most consistent with a simple bimolecular reversible binding process (Figure 4F).

**Figure 5.**
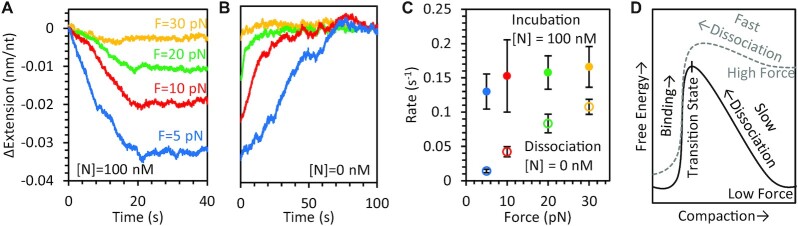
Force dependence of NTD binding. The extension of the ssDNA when incubated with 100 nM NTD only N protein (**A**) and in the absence of free protein (**B**) are plotted over time. Unlike full-length protein, the binding of the NTD occurs in a single step and is fully reversible. The degree of compaction is increased at lower ssDNA tensions. (**C**) The average rates (*N* = 3 experimental replicates for each condition) of ssDNA compaction during incubation (solid circles) do not vary significantly with force, but the rate of decompaction due to protein dissociation (open circles) increases with applied force. (**D**) An idealized energy landscape of an ssDNA binding site is plotted showing two stable states, protein free (less compact, left) and protein bound (more compact, right), separated by an energy barrier, whose height determines the rate of protein binding and dissociation. For simplicity, the landscape (solid black) is plotted at protein concentration equal to the dissociation constant, such that the free energies of the bound and unbound states are equal. Applying force tilts the energy landscape (dotted gray) proportional to compaction (work equals force times displacement), favoring the protein-free, uncompacted state. Our measured rates indicate the transition state is closer to the unbound state, such that rate of binding is less dependent on force (initial binding does not require ssDNA deformation, e.g. electrostatic binding) than the rate of dissociation (removing protein releases ssDNA length for extension).

Due to the reduced compaction caused by the NTD only variant, it was also possible to fully relieve tension on the substrate and measure compaction at lower forces (Figure 5A). We find that the binding of the NTD only variant reduces ssDNA extension by a greater degree at lower forces, but that the compaction remains fully reversible (Figure 5B). Since the NTD variant binds in a one step process, we were also able to measure directly the rates of N protein binding to and dissociation from the ssDNA substrate over all forces (Figure 5C). We found that the rates of compaction induced by protein binding were force independent, while higher force significantly increased the rate of decompaction induced by protein dissociation. Since protein binding reduces ssDNA extension in opposition to the force applied by the optical tweezers, force should decrease protein binding affinity. Thus, these measured rate dependencies are consistent with an energy landscape in which the transition state (the kinetic barrier that controls the rates of binding and dissociation) is closer to the unbound, uncompacted state than to the bound, compacted state. Specifically, the rate-limiting event in NTD/ssDNA binding is diffusion-limited and does not involve significant ssDNA compaction. In contrast, the dissociation of the protein immediately releases almost the whole length of ssDNA, which was compacted upon N protein binding, leading to a significant facilitation of the dissociation rate by applied force.

### CTD protein variants compact DNA more slowly than full-length N protein but to the same extent

**Figure 6.**
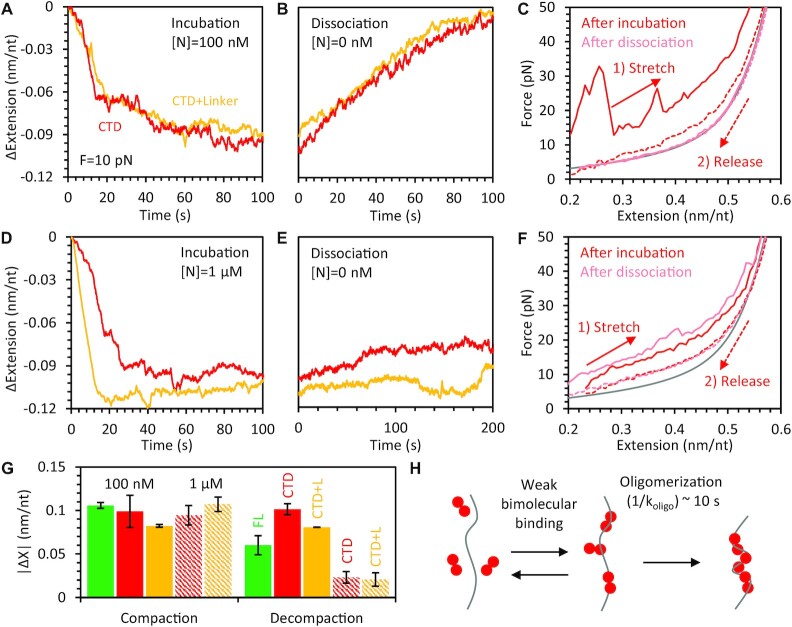
Compaction of ssDNA by CTD variant proteins. (**A**) Incubation of ssDNA held at 10 pN with 100 nM N protein is repeated with CTD (red in all panels) and CTD + L (yellow in all panels) protein variants. Compaction occurs over a longer timescale and with less discrete compaction steps compared to FL N protein, but with similar final compaction amplitude. (**B**) Removal of free protein results in slow but complete dissociation of protein as the ssDNA returns to its original conformation. (**C**) Stretching the ssDNA after incubation (red) with CTD and dissociation (light red) shows large compacting structures after incubation and complete protein removal after dissociation. (D-F) Experiments are repeated with increased 1 μM concentration of N protein CTD variants. Compaction at the start of incubation is faster (**D**) and bound protein and compact structures remain after removal of free protein, as observed in the lack of decompaction (**E**) and the remaining compaction in the stretch curve (**F**). (**G**) The amplitude of final compaction after N protein incubation and decompaction in the absence of free protein is averaged over *N* = 3 experiments for each CTD variant and concentration condition and compared to FL protein results (Figure 2). Solid bars correspond to 100 nM N protein concentration and hatched bars correspond to 1 μM N protein variants. (**H**) Model of CTD dimers binding to and oligomerizing on a nucleic acid substrate as a two-step process. Initial bimolecular binding is weaker than for protein containing NTD, limiting the rate of oligomerization at lower protein concentrations. Sufficiently large oligomers are effectively irreversibly bound on the timescale of our experiments (up to 1000 s).

Binding and compaction experiments were repeated using N protein truncations containing just the CTD and the C-tail, with or without the linker. Again, we simply denote these variants as CTD(+L), but the disordered C terminus is also included, which can interact with the structured domain (26). Both CTD variants fully compacted the ssDNA during incubation. However, the kinetic profile matched neither the single-phase exponential of the NTD variants nor the two distinct compaction steps separated by a measurable pause of the FL protein. Instead, compaction occurred in a monotonically increasing but highly stochastic manner with compaction occurring over a longer period of time (Figure 6A). Additionally, when free protein was removed from the sample, the ssDNA eventually fully decompacted, returning to a protein free ssDNA state (Figure 6B). Though again, the kinetic profile did not follow a single exponential decay, as would be expected for individual proteins independently dissociating at a fixed rate. Instead decompaction proceeded nearly linearly over time. The FEC obtained after incubation confirmed the CTD variants initially formed highly compact structures on the ssDNA that were disrupted at high force (Figure 6C). The FEC obtained after 200 s of dissociation also confirmed that all protein and compacted structures were removed from the ssDNA.

As previous research has indicated that the NTD strongly binds single-stranded nucleic acid substrates (12,14,16,17), it is probable that CTD variants missing the NTD will have reduced binding affinity to the ssDNA substrate used here. This in turn could affect the degree of protein saturation on the ssDNA (i.e. whether all possible binding sites on the ssDNA are occupied) and local protein density on the substrate. Since interprotein interactions, such as oligomerization, are typically enhanced when proteins are in close contact, we repeated the compaction experiments again with a 10X increase in protein concentration (1 μM), to see if it would affect the CTD’s ability to form large oligomers. As expected, the increased protein concentration resulted in faster ssDNA compaction during incubation (Figure 6D). However, the equilibrium degree of compaction at the end of incubation was not significantly increased over the 100 nM protein concentration experiments. When free protein was removed from the system, little decompaction was observed (Figure 6E), indicating the compacted structures formed were stable for much longer than the timescale of our experiments (several minutes). FECs obtained both directly after incubation and after dissociation both showed a large degree of compaction (Figure 6F).

Whereas the kinetics of compaction are markedly different for the FL N protein and the CTD variants, the final degree of compaction at the end of protein incubation is similar (Figure 6G). Our results with the N protein CTD variants are most consistent with a multistep binding process, such as bimolecular binding between free CTD dimers and the ssDNA substrate with reduced affinity compared to FL protein, followed by protein oligomerization that depends on high protein density along the substrate (Figure 6H).

### N protein does not compact dsDNA, but imposes torsional restraints on strand unwinding

**Figure 7.**
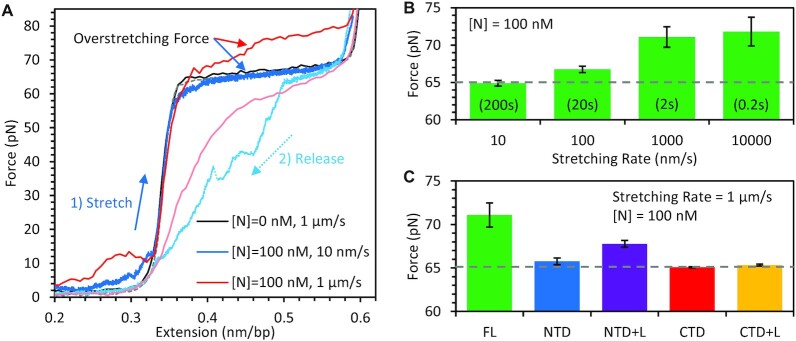
Binding of N protein to dsDNA. (**A**) Stretching protein-free dsDNA (black) yields a force dependence well described by an idealized wormlike chain polymer with contour length of 0.34 nm/bp before undergoing overstretching at a force of >60 pN (sequence and salt dependent), which disrupts base pairing and stacking concurrent with DNA strands unwinding and lengthening by ∼70%. Releasing the dsDNA back to its original extension (dashed gray) results in minimal hysteresis as the disrupted base pairing, stacking, and strand winding of B DNA are quickly restored when force is decreased. When the dsDNA is stretched after incubation with 100 nM N protein for 100 s, weak dsDNA aggregation/looping is pulled apart at low force but no change in the contour length of dsDNA is observed. However, N protein binding leads to an increased overstretching force, which is more pronounced for fast stretching (1 μm/s, red) versus slow stretching (10 nm/s, blue). Both release curves (dashed lines) also show substantial hysteresis. (**B**) The average (N = 5 for each condition) force of the ∼2 μm long overstretching transition as a function of pulling rate shows a substantial increase when the DNA is overstretched on a timescale of <10 s, as compared to the average experimentally observed overstretching force for this DNA construct stretched in 150 mM NaCl in the absence of protein (dashed gray line). (**C**) The average (*N* = 3 for each truncated variant) overstretching force for fast stretching (1 μm/s) of dsDNA after incubation with 100 nM N protein is reduced for truncated protein variants (more similar to protein-free dsDNA, dashed gray line), indicating weaker or no torsional constraint imposed on dsDNA by these protein variants.

Whereas our experiments primarily focus on N protein binding ssDNA, as SARS-CoV-2 viral particles contain ssRNA, the viral genome can form regions of secondary structure such as hairpins. These base paired regions of RNA tend to produce an A-form helix, which is locally stiff with a persistence length of *p* ≈ 60 nm (39). B-form dsDNA is similarly stiffened (*p* ≈ 50 nm) compared to more flexible ssRNA and ssDNA (*p* ≈ 0.08 nm) (33). Since N protein's binding conformation on ssDNA requires substantial substrate compaction, this binding mode is likely inhibited on a stiffer substrate. To test the ability of N protein to bind a helical NA, we repeated our binding experiments using a dsDNA substrate. When incubating a straightened dsDNA substrate (*F* > 5 pN) with N protein, we did not observe significant compaction of the substrate (Figure 7), unlike that observed for ssDNA (Figure 2). This observation also presents opportunities for future experiments where dsDNA handles can be used to tether other biomolecules of interest. For example, this would allow direct observation of N protein interactions with RNA structures present in the SARS-CoV-2 genome, similar to our previous studies of HIV-1 nucleocapsid protein and RNA hairpins (40,41). However, we do see evidence of N protein binding. First, when the dsDNA was held at approximately half its extended length before stretching in the presence of N protein, we observed a slight reduction in dsDNA extension only at low forces (<10 pN) that was quickly removed during stretching. This is consistent with N protein temporarily stabilizing loops of dsDNA that naturally form when its two ends are held in close proximity. In contrast, much higher forces (>50 pN) were required to decompact ssDNA in the presence of N protein (Figure 2C). Once these weak, low force loops were removed, the dsDNA FEC exhibited the same extended length as protein free dsDNA, as described by the worm like chain (WLC) polymer model.

Further evidence of N protein binding was observed when the dsDNA substrate undergoes an overstretching transition (65 pN for 150 mM NaCl buffer), in which base pairing and base stacking are disrupted, the two strands of B-form DNA unwind, and the substrate extends by ∼70% of its length over a small increase in force. dsRNA undergoes an equivalent transition at a slightly reduced force (42). The exact force at which the helix overstretches is determined by the ability of the two strands to unwind from one another, enabled by our system's lack of torsional constraints due to labeling only one strand of the dsDNA molecule. Both strand unwinding during overstretching and strand rewinding upon release occur quickly, such that the overstretching transition is in equilibrium. That is, the stretch and release curves overlap with minimal hysteresis (Figure 7A black). Interestingly, when the dsDNA was extended quickly (1 μm/s, Figure 7A red) in the presence of N protein, we observed an apparent increase in the overstretching force. This increase was not present for slow stretching (10 nm/s, Figure 7A blue). This rate-dependent increase in force indicates that N protein can induce torsional constraint on the dsDNA strand winding, as was previously observed for the dsDNA torsionally constrained either by its attachment (43), or by the bound protein (44). Consistent with this hypothesis, the fast release of dsDNA tension in the presence of N protein leads to lower force of the overstretching transition, as the two strands take longer to rewind back into the B DNA. This could result from N protein simultaneously binding both strands of the dsDNA, inhibiting their unwinding or rewinding relative to each other.

By stretching dsDNA in the presence of N protein at different rates, we found that overstretching over a timescale of less than ∼10 s resulted in increased overstretching force (Figure 7B), thus defining the lifetime of N protein mediated torsional constraint. The simplest interpretation is that individual N proteins (or protein dimers) remain bound to both strands simultaneously, possibly through multiple binding domains, for only ∼10 s, after which at least one strand is released allowing unwinding of the strands. This is a faster timescale than the ∼50 s required for full dissociation of N protein from the ssDNA substrate held at 10 pN (Figure 2B). Thus, this shorter timescale of torsional constraint is reflective of unbinding from just one strand/site, not full dissociation, consistent with high force stretching decompacting the ssDNA-N protein complex but not triggering full protein dissociation. Overall, our results show that N protein binds dsDNA, forming contacts with both strands, but has reduced affinity as compared to ssDNA binding.

We repeated these dsDNA stretching experiments with the truncated N protein variants (Figure 7C). Compared to the full-length protein, all variants showed reduced ability to inhibit dsDNA strand unwinding. This could indicate either reduced binding affinity (less of the substrate is covered with protein) or inability of the individual N domains to bind both DNA strands simultaneously. The only variant that significantly increased the overstretch force was the NTD with linker variant, suggesting that the NTD and the central linker of N protein can work as two independent units simultaneously binding two DNA strands.

## DISCUSSION

### Optical tweezers allow direct measurements of compaction functions of N protein

SARS-CoV-2 N protein has two major functions in the viral life cycle (28): packaging the nearly 30 kb vRNA genome into a ∼80 nm diameter viral particle (2,10) and facilitation of transcription of that genome into mRNA for protein synthesis (4). Both functions are affected by the N protein ability to bind RNA and its tendency to form aggregates. The aggregation of N protein with RNA has been observed as liquid-liquid phase separation (LLPS) with results dependent on the NA substrate (23,45–48) and the phosphorylation of N protein (28,49). The structures of these aggregates have also been examined using fluorescence microscopy (23,46,47). Attempts have been made to identify a vRNA packaging signal that induces preferential packaging of SARS-CoV-2 RNA by measuring binding of N protein to specific RNAs (32,46,47,50). However, *in vitro* experiments have not measured preferential binding (23), suggesting RNA binding by N protein may be primarily non-specific, and that packaging is sensitive to the type of RNA–protein aggregate formed rather than enhanced binding to a specific packaging signal. This work expands upon these bulk studies by utilizing optical tweezers to isolate a single NA substrate, preventing the commonly observed inter-substrate aggregation. Instead, we observe the intra-substrate aggregation that causes compaction necessary for packaging in real time.

### Separation of function observed in nucleic acid binding of N protein NTD, CTD and linker domains

Our kinetic profiles of ssDNA compaction by FL N protein show two clear steps (Figure 2), the first of which strongly resembles the binding activity of the NTD in isolation (Figure 4). Specifically, the NTD variants bind the substrate at the same fast, diffusion limited rate (∼10^6^ M^–1^ s^–1^) as the full-length protein, indicating the rest of the protein, specifically the CTD, does not substantially contribute to initial binding. Furthermore, the NTD’s rate of binding is not strongly dependent on the substrate tension (Figure 5) but is effectively screened at higher salt concentrations (Figure 3), indicating electrostatically driven binding without strong initial NA compaction. Finally, the single-phase rate of both NTD variants’ binding and dissociation and the full dissociation of bound protein indicate that the NTD binds ssDNA in a non-cooperative and fully reversible manner. The NTD acting as the primary NA binding site and in a non-cooperative, electrostatic manner is consistent with previous studies (46,47). Interestingly, since the NTD variants lack the primary dimerization interface of the CTD, they are presumably monomeric in solution. If the full-length protein is primarily dimeric in solution, however, each dimeric unit has two NTDs which could enable additional ssDNA compaction through simultaneous binding of these two domains. However, we do not observe this. Instead, it appears that each NTD binds independently, inducing the same ssDNA compaction as two monomeric NTDs, potentially because the disordered linker that decouples the NTD and CTD also effectively prevents coordination of the dimer's two NTDs.

The dramatic difference in the degree of compaction exhibited by the NTD variants with and without the unstructured central linker indicates that the linker is also critical to the proteins’ binding conformation. While the globular NTD must compact ssDNA through bending within the cationic groove (23), the linker with its serine-arginine (SR) rich region also contributes to stronger NTD + L binding with ssDNA compaction, while preserving fast on/off kinetics and non-cooperative binding. This may result from NTD to linker intra-molecular binding (51) concurrent with both binding sites constraining the ssDNA, leading to its stronger compaction. A direct interaction between the linker and NA would explain the dramatic changes in protein function caused by linker phosphorylation (28). In particular, the linker SR region, the N protein's primary phosphorylation site, could associate with NAs via electrostatic interactions (27). Also, naturally occurring mutations at this site, (S202R) and (R203M), were associated with a 50-fold increase in vRNA packaging (50). Thus, the exclusion of the linker from N protein in our experiments may mimic SR site phosphorylation, which neutralizes arginine's electrostatic NA interactions. As our *in vitro* experiments utilize proteins expressed in *E. coli*, the behavior we observe should be consistent with the unphosphorylated form of N protein. Thus, the increased NA compaction we observe may be partially responsible for differences in protein-NA aggregate formation, with the unphosphorylated protein producing rigid, branched, immobile, and slowly growing aggregates, whereas the phosphorylated protein forms liquid like, high internal mobility, and fast growing aggregates (27,28).

While initial binding of the full-length N protein resembles the NTD in isolation, the secondary compaction step requires the CTD. This sudden, fast transition to a more compacted state is indicative of a highly cooperative process, in which the entire strand is compacted in a single step. However, this fast transition requires a fully protein-saturated NA substrate, as lower free protein concentrations, which require longer timescales to fill all available binding sites, result in less discrete compaction steps (Figure 3). Thus, while the substrate is eventually highly compacted at the end of incubation, localized patches of protein must be compacting concurrently as free protein fills in the binding lattice, resulting in a smooth curve without distinct compaction events. Similarly, for ssDNA incubation with 100 nM CTD variants (Figure 6A), compaction occurs continuously over a long timescale, without the pause between initial and secondary compaction observed for FL protein experiments. However, increasing protein concentration results in immediate full compaction (Figure 6D), suggesting lower binding affinity limits the rate of the compaction process. The compacted ssDNA–protein complex also inhibits dissociation, with incubation with 100 nM CTD protein resulting in slow, linear-like dissociation, more consistent with breakdown of protein aggregate from the ends than with independent dissociation of individual proteins and incubation with a saturating 1 μM concentration resulting in negligible decompaction. These results are consistent with N protein oligomerizing along the nucleic acid substrate, due to increased local concentration along the substrate compared to bulk conditions, and this activity requiring the CTD, consistent with previous observations of CTD-mediated oligomerization (46,47). Interestingly, the total extent of compaction observed for ssDNA saturated with FL and CTD N protein is equal, suggesting while the presence of the NTD impacts the pathway and kinetics of ssDNA compaction, the final compacted state we observed with an applied force ∼10 pN did not require the presence of the NTD.

N protein forming a condensed bead-like structure in combination with vRNA has been observed both *in vitro* (28) and in the virion (52,53). Similar structures were previously observed for other coronaviruses (20,54). However, even the most recent CryoEM structures with spatial resolution of ∼1 nm are insufficient to clearly follow protein arrangement and the path of RNA within the ribonucleoprotein (RNP) complex. One model proposes that the ∼30 kb long vRNA is organized into three levels of compaction (53). The ∼80 nm diameter virion contains ∼40 bead structures with approximate diameter of 15 nm. Each of these beads is composed of 5–6 N protein dimers arranged in a ‘G’ like shape. Finally, the vRNA is wrapped around each ‘L’ shaped N protein dimer, with 130–160 nt RNA occupied per dimer. While the minimum extension imposed upon the tethered ssDNA by our optical trap would prevent the formation of these condensed beads, the compaction we do measure could be the initial step of the nucleic acid substrate wrapping around protein dimers followed by some dimer-dimer interactions. This beaded RNP structure would require interdimer contacts, which have not been resolved in detail from available structures (52,53). According to the structural modeling of full length N protein dimers based on small-angle X-ray scattering the NTDs do not directly interact with each other or the CTD (51).

Alternatively, the beaded RNP structure could be maintained primarily by strong CTD–CTD interactions, with contributions from weaker NTD-linker interdimer contacts. Indeed, other studies have found the CTD itself can aggregate RNA (46,47), and previous models of homologous N proteins from other coronaviruses have predicted CTD based structures (13,20,21,54). Based on available structures of the CTD of SARS-CoV-1 and Mouse Hepatitis Virus (13,21), it is proposed that N protein forms octamers through tetramerization of CTD mediated dimers and that these octamers weakly stack with one another to form a helical filament. The NTDs would then be arranged around the periphery of the CTD stabilized helix, such that the vRNA could wrap around the cationic groove formed by the CTD core and adjacent NTDs. Assuming this RNP arrangement, we estimate the degree of NA compaction from a purely geometric perspective (Supplemental Figure S3). The contour lengths of bare NA without secondary structure (*L*) and of the helical RNP complex (*L*′) are related through the radius (*R*) and helical pitch (*ρ*) of the helix as:

(1) \begin{eqnarray*}\frac{L}{{L^{\prime}}} = {\left[ {{{\left( {\frac{{2\pi R}}{\rho }} \right)}}^2 + 1} \right]}^{1/2}\end{eqnarray*}

Using the parameters derived from (21) of *R* = 4.5 nm and *ρ* = 7 nm, we calculate a compaction ratio of (*L/L*′) ≈ 4. That is, the helical RNP is four times shorter than the original protein-free NA substrate. This is much stronger compaction than the ∼25% length reduction we observe under a tension of 10 pN, though our experimental FECs show even greater compaction at lower forces, so that applied force may impact the NA’s path as it wraps around the protein oligomer. The equal degrees of compaction we observed for both FL and CTD only N proteins suggest that the NTD is not directly involved in interprotein interaction and that the multimerization surfaces needed for forming the compacted structure reside solely in the CTD. However, our data cannot definitively rule out alternative ways of RNP arrangement discussed above. It is also possible that multiple pathways of NA aggregation by N protein exist, dependent on the NA substrate, protein modification, and external conditions. For example, the beaded RNP structure of non-phosphorylated N protein with vRNA found inside the virion may require NTD-linker contacts while alternative RNP structure formed by phosphorylated N protein with any non-specific NA may involve CTD–CTD only contacts.

**Figure 8.**
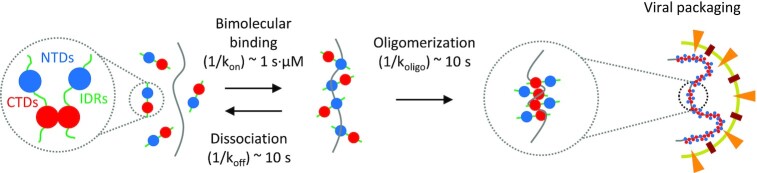
Model of multistep process of N protein binding and packaging vRNA. N protein is abundant in the host cell, existing primarily as a dimer, mediated by contacts between the CTDs with the NTDs separated by the linker IDRs. When N protein encounters viral RNA, bimolecular binding occurs, primarily mediated by the NTDs. At micromolar concentrations, the RNA substrate is saturated on a second time scale (provided enough protein is present to occupy all available binding sites), with each individual bound protein remaining on the RNA substrate for tens of seconds. While the RNA is saturated with N protein, the local concentration of protein is extremely high, allowing for higher order oligomeric states to form. The structure of the RNA-protein complex is determined by interactions involving CTDs of neighboring proteins. Once in this oligomeric state, binding of the protein oligomers is very stable, preventing individual proteins from dissociating from the substrate and the RNA from decompacting. The nearly 30 kb viral RNA genome (∼16 μm extended length) is compacted over 100× in order to fit inside the ∼80 nm diameter viral particle.

Our results, which separate the binding properties and kinetics of the N protein NTD and CTD, suggest a multistep process through which N protein binds and packages viral RNA (Figure 8). N protein is highly expressed in the host cell, resulting in >μM concentrations of free protein that form CTD-mediated dimers. Any available vRNA substrate is immediately bound by N protein through the NTDs and linkers with high affinity in a diffusion-limited manner. This binding is primarily electrostatic, non-cooperative, fully reversible, and requires minimal substrate deformation. Individual proteins may remain bound to the substrate for timescales on the order of 10 s, but over time neighboring proteins multimerize, stabilizing binding and inhibiting protein dissociation. This process could occur for the entire ∼30 knt vRNA at once, or individual large regions could independently begin compaction around localized nucleation events and eventually merge such that the entire viral genome is compacted. This higher order protein oligomerization is mediated primarily by the CTD, and is highly cooperative such that a fully saturated substrate can be fully compacted within 10 s. Of course, the degree of ssDNA–protein complex compaction observed in this work is more modest than vRNA-protein compaction inside the ∼80 nm diameter viral particle.

## DATA AVAILABILITY

Data are available upon request to the corresponding author.

## Supplementary Material

gkac1179_Supplemental_File
