# Supplementary material for: Structural domains of SARS-CoV-2 nucleocapsid protein coordinate to compact long nucleic acid substrates
Source: Nucleic Acids Res. 2022 Dec 19;51(1):290–303. doi: 10.1093/nar/gkac1179 (PMC9841419; doi:10.1093/nar/gkac1179)
Supplement: gkac1179_Supplemental_File [file gkac1179_supplemental_file.pdf]

Supplemental Materials

A

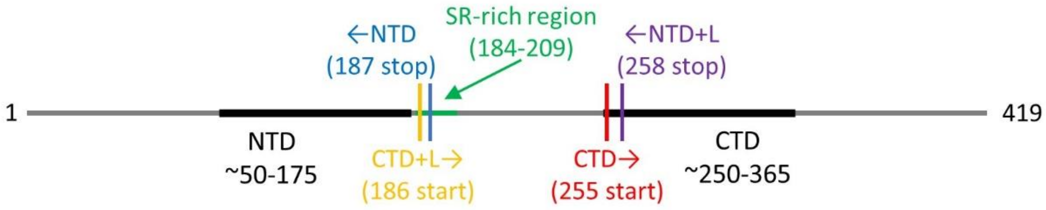

B

```
Polyhistidine tag        1        10         20         30         40
MGSSHHHHHHSSGLEVLFQGPAS MSDNGPQNQR NAPRITFGGP SDSTGSNQNG ERSGARSKQR
          50         60         70         80         90        100
  RPQGLPNNTA SWFTALTQHG KEDLKFPRGQ GVPINTNSSP DDQIGYYRRA TRRIRGGDGK
         110        120        130        140        150        160
  MKDLSPRWYF YYLGTGPEAG LPYGANKDGI IWVATEGALN TPKDHIGTRN PANNAAIVLQ
         170        180        190        200        210        220
  LPQGTTLPKG FYAEGSRGGS QASSRSSSRS RNSSRNSTPG SSRGTSPARM AGNGGDAALA
         230        240        250        260        270        280
  LLLLDRLNQL ESKMSGKGQQ QQGQTVTKKS AAEASKKPRQ KRTATKAYNV TQAFGRRGPE
         290        300        310        320        330        340
  QTQGNFGDQE LIRQGTDYKH WPQIAQFAPS ASAFFGMSRI GMEVTPSGTW LTYTGAIKLD
         350        360        370        380        390        400
  DKDPNFKDQV ILLNKHIDAY KTFPPTEPKK DKKKKADETQ ALPQRQKKQQ TVTLLPAADL
         410        419
  DDFSKQLQQS MSSADSTQA
```

Supplementary Figure 1. N protein sequence. (A) Diagram shows locations of protein truncations relative to residues typically associated with structured domains (NTD and CTD) and serine rich region of IDR linker. (B) Full length (FL) N protein (aa 1-419) was expressed with a polyhistidine tag, which was cleaved from the protein during purification. For NTD only variants, the protein was truncated with a stop codon after residue 187 (NTD, blue) or 258 (NTD+linker, purple). For the CTD only variants, the protein starts at residue 186 (CTD+linker, yellow) or 255 (CTD, red).

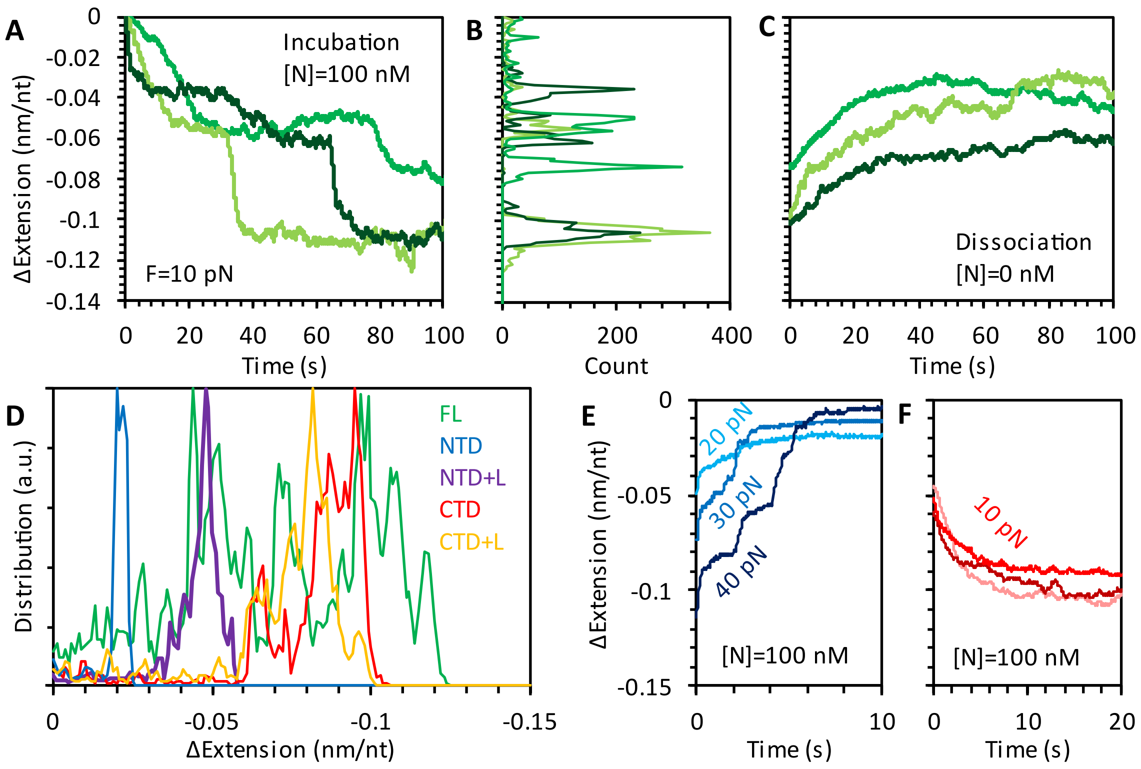

Supplementary Figure 2: Comparison of experimental replicates and protein variants. (A) Three experimental replicates are shown for FL N protein compacting ssDNA (Figure 2). Exact kinetic profile between experiments is inconsistent, with variable time delays between the initial compaction at the beginning of incubation (t=0) and subsequent compaction events, but every profile shows multistep compaction, as evidenced by two or more peaks in instantaneous extension histogram (B). (C) Each experiment shows partial decompaction due to protein dissociation, with variable amplitude but consistent rate. (D) Summation of extension histograms for all 100 nM FL N protein experiments (N=10), shows two main peaks around 0.05 and 0.1 nm/nt compaction, with a much smaller peak in between for experiments that had an additional pause between initial and final compaction. In comparison, for truncated N protein variants (N=3 each), there are not clearly separated multiple peaks. For the NTD only variants, which exhibit a simple one-step bimolecular binding process (Figure 3), the ssDNA quickly compacts without pausing before equilibrating, resulting in a single sharp peak. For CTD only variants (Figure 5), the ssDNA extension decreases in a monotonic but chaotic pattern, resulting in a poorly defined peak near its final extension value. (E) When the tension applied to ssDNA equilibrated with 100 nM FL N protein at low force is suddenly increased, the ssDNA extends in multiple variable steps with unreproducible shape between experimental replicates, similar to stretching the ssDNA to full extension (Figure 5F). (F) In contrast, reducing the tension back to 10 pN allows the ssDNA-N protein complex to recompact in a single step process with reproducible shape (three replicates shown).

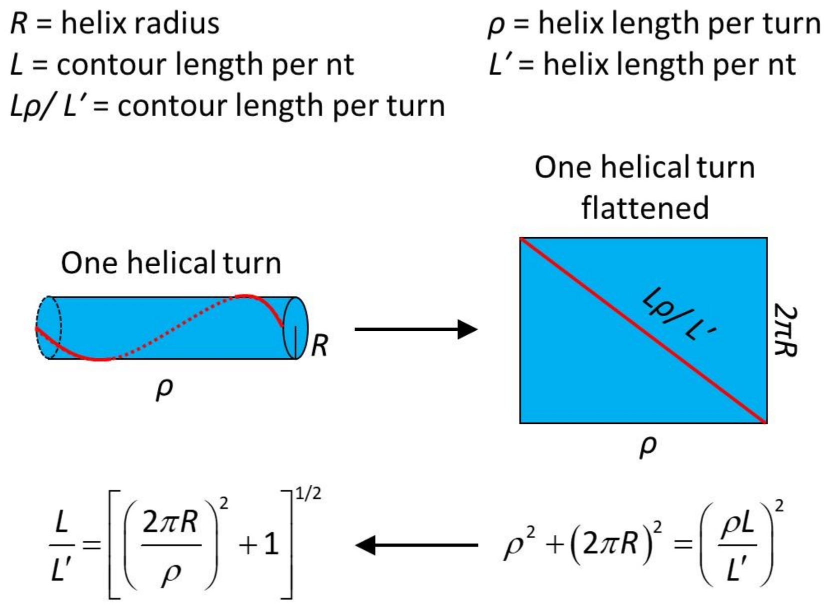

Supplementary Figure 3: Geometric model of a helical RNP. One full turn of helix is represented as a nucleic acid (NA) substrate wrapped around a cylinder of length *ρ* and radius *R* (left). The cylinder is flattened into a 2-dimensional rectangle (right), where the NA stretches from one opposing corner to the other. The length of NA substrate per helical turn (*Lρ/L′*) can be related to the length and circumference (2*πR*) of the cylinder by the Pythagorean theorem. This expression is algebraically rearranged to solve for the ratio of the contour length of the original RNA/DNA substrate (*L*) to the length of the helical RNP (*L′*) as in Equation 1.
